# MicroRNA dysregulation in multiple sclerosis

**DOI:** 10.3389/fgene.2012.00311

**Published:** 2013-01-22

**Authors:** Omar de Faria Jr, Craig S. Moore, Timothy E. Kennedy, Jack P. Antel, Amit Bar-Or, Ajit S. Dhaunchak

**Affiliations:** ^1^Department of Neurology and Neurosurgery, The Montreal Neurological Institute and Hospital, McGill University Health Centre, McGill UniversityMontreal, QC, Canada; ^2^Program in NeuroEngineering, McGill UniversityMontreal, QC, Canada; ^3^Neuroimmunology Unit, Montreal Neurological Institute and Hospital, McGill University Health Centre, McGill UniversityMontreal, QC, Canada

**Keywords:** miRNA, multiple sclerosis, myelin, MS lesions, immune system

## Abstract

Multiple sclerosis (MS) is a chronic inflammatory disease characterized by central nervous system (CNS) demyelination and axonal degeneration. Although the cause of MS is still unknown, it is widely accepted that novel drug targets need to focus on both decreasing inflammation and promoting CNS repair. In MS and experimental autoimmune encephalomyelitis, non-coding small microRNAs (miRNAs) are dysregulated in the immune system and CNS. Since individual miRNAs are able to down-regulate multiple targeted mRNA transcripts, even minor changes in miRNA expression may lead to significant alterations in gene expression. Herein, we review miRNA signatures reported in CNS tissue and immune cells of MS patients and consider how altered miRNA expression may influence MS pathology.

## INTRODUCTION

Multiple sclerosis (MS) is a chronic demyelinating neurodegenerative disease of the central nervous system (CNS) with an unknown etiology. Several lines of evidence suggest that developing this autoimmune disease depends on both environmental and genetic factors, which is supported by genome-wide association studies. Although the exact pathogenesis of MS remains to be fully elucidated, the pathology of MS suggests an autoimmune etiology and includes infiltration of T cells, B cells, and macrophages in active MS brain lesions. The immune attack in the CNS is mediated by autoreactive T cells that enter through a disrupted blood–brain barrier (BBB) and attack oligodendrocytes and myelin. While CD4^+^ T cells are considered essential in the initial stages of CNS inflammation, both adaptive and innate (microglia/macrophages) immune mechanisms contribute to inflammation and tissue injury (Prat and Antel, 2005; Becher et al., 2006). In the periphery and CNS, altered cytokine profiles (e.g., TNF-α, IFN-γ, IL-17, IL-6, and IL-18) contribute to the pathological features of MS, such as demyelination, loss of oligodendrocytes, and axonal degeneration (Lucchinetti et al., 2000; Bjartmar et al., 2003; Imitola et al., 2005). The balance between Th1 (pro-inflammatory) and Th2 (anti-inflammatory) mechanisms contributes to injury, but also repair. Several cytokines (including Th1 and Th2) are implicated in the survival and differentiation of neurons, oligodendrocytes, and oligodendrocyte progenitor cells (OPCs). Given the contribution of cytokines and chemokines to CNS injury and repair, tight transcriptional and translational regulation of these molecules is required. In recent years, microRNAs (miRNAs) have emerged as important regulators of cytokine and growth factor expression, and have been suggested as disease biomarkers and targets of therapy.

MicroRNAs are evolutionarily conserved, short non-coding RNAs that regulate gene expression in health and disease, during development, immune system activation, neurogenesis, and myelin formation in the CNS. Several studies have implicated the disruption of miRNA function in human diseases. In MS, miRNA profiles are altered within CNS lesions and in the immune system, and affect gene expression in many cell types involved in the disease. A global consideration of miRNA dysregulation and the resulting alterations in gene expression may provide valuable insights into the pathophysiology of MS and reveal new alternatives for early diagnosis and treatment.

## miRNA DYSREGULATION IN THE IMMUNE SYSTEM

In the human immune system, miRNAs play an important role in modulating innate immune responses against bacteria, viruses, and other pathogens. These inflammatory responses of immune cells against invading pathogens must be efficiently and tightly regulated. miRNAs also influence the development and regulation of the adaptive immune system. Biological activities of T and B cells are influenced by specific miRNAs. More specifically, miRNAs regulate B and T cell development and differentiation, in addition to pro-inflammatory responses mediated by T_reg_ cells. Given the complex pathobiology of MS and the involvement of both innate and adaptive immunity, examining miRNA profiles in specific immune cell subsets may reveal potential novel biomarkers and further elucidate underlying mechanisms.

MicroRNAs have been identified that are differentially expressed between MS disease subtypes and healthy individuals. Several of these studies have used high-throughput miRNA profiling to identify key miRNAs unique to different aspects of disease. RNA sources have included whole blood, serum, peripheral blood mononuclear cells (PBMCs), and lymphocytes. Significantly decreased levels of miR-17 and miR-20a have been detected in whole blood in all MS subtypes compared with healthy controls. Notably, miR-17 and miR-20a block genes involved in T cell activation (Cox et al., 2010). Also in whole blood, 165 miRNAs were reported to be significantly up- or down-regulated; with miR-145 identified as a highly sensitive, specific, and accurate miRNA (Keller et al., 2009). Additional studies have isolated PBMCs from MS patients during both relapses and remissions, and have identified differential expression of miR-18b, miR-493, and miR-599, which have been implicated in interleukin and wnt signaling (Otaegui et al., 2009). miR-21, miR-146a, and miR-146b have also been reported to be significantly increased in whole PBMCs from relapsing-remitting multiple sclerosis (RRMS) patients compared with controls (Fenoglio et al., 2011).

**FIGURE 1:**
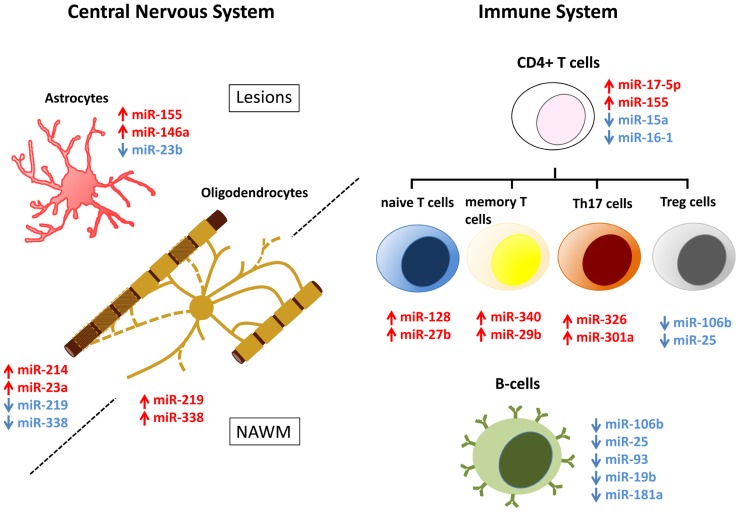
**MicroRNA (miRNA) dysregulation in CNS lesions and immune cells of MS patients**. miRNAs that are up- (red) and down-regulated (blue) in CNS lesions, NAWM, or immune system of MS patients have been assigned to specific cell types. Dysregulation is associated with astrocytes and oligodendrocytes in the CNS and naïve T cells, memory T cells, Th17 cells, T_reg_ cells, and B cells in the immune system. Note that while in most cases miRNA dysregulation has been specifically detected in the mentioned cell types, miRNAs that are here assigned to oligodendrocytes, were done so exclusively on the basis of their relevance to normal oligodendrocyte biology.

The importance of correlating disease-relevant functions with altered miRNA levels has prompted others to profile individual miRNAs within immune cell subsets (**Figure 1**). In CD4^+^ T cells, higher levels of miR-17-5p was found in RRMS patients, leading to down-regulation of targeted genes (Lindberg et al., 2010). In contrast, miR-15a and miR-16-1 are down-regulated in CD4^+^ T cells from RRMS patients (Lorenzi et al., 2012). These authors suggest that elevated levels of bcl-2 in CD4^+^ T cells from MS patients result from decreased levels of miR-15a and miR-16-1, which target this potent anti-apoptotic gene (Lorenzi et al., 2012). miR-128 and miR-27b are significantly elevated in naïve T cells from MS patients compared with healthy controls, while miR-340 was increased in memory T cells (Guerau-de-Arellano et al., 2011). Functionally, changes in these specific miRNAs inhibit Th2 cell differentiation by inhibiting B lymphoma Mo-MLV insertion region 1 homolog (BMI1) and IL-4 expression, providing evidence that miRNAs influence T cell polarization. A recent study has identified miR-29b as an IFN-γ-inducible miRNA in CD4^+^ memory T cells, which acts in a negative feedback loop to control Th1 cell bias by inhibiting T-bet and IFN-γ transcription. In MS patients, the increase in miR-29b suggests a dysregulation of this feedback loop and an important factor that can bias Th1 cell differentiation (Smith et al., 2012).

Th17 cells are a subset of effector T cells that contribute to inflammation and tissue damage in autoimmune diseases, including MS. Th17 cell differentiation is controlled by several cytokines and growth factors, yet role(s) for miRNAs have not been identified. miR-326 is significantly increased in peripheral blood leukocytes of MS patients, and is prominently expressed by CD4^+^/IL17a^+^ T cells compared to other T cell and non-T cell populations (Du et al., 2009). miR-326 targets Ets-1, a negative regulator of T cell differentiation, thereby promoting differentiation (Moisan et al., 2007). In experimental autoimmune encephalomyelitis (EAE), an animal model of neuroinflammation, by using miRNA mimics and inhibitors *in vivo* it was shown that overexpression of miR-326 increased Th17 cell number and disease severity, while decreasing miR-326 expression resulted in relatively mild EAE accompanied by fewer Th17 cells (Du et al., 2009). Also in the EAE model, miR-301a regulates Th17-mediated immune responses by targeting the IL-6/23-STAT3 pathway. Studies performed *in vivo* have demonstrated that modulating levels of miR-301a can significantly alter EAE severity (Mycko et al., 2012). miR-155, an inflammatory-promoting miRNA that targets suppressor of cytokine signaling (SOCS), is elevated in CD4^+^ T cells in EAE. Targeting miR-155 using antisense oligonucleotides, resulted in decreased EAE severity when administered both prior to and after the onset of clinical scores (Murugaiyan et al., 2011). Mice lacking miR-155 (mir-155^-^^/^^-^) have reduced EAE disease severity accompanied by less CNS inflammation and decreased Th1 and Th17 responses (O’Connell et al., 2010).

In B lymphocytes isolated from untreated RRMS patients, the levels of 49 miRNAs are reported to be significantly decreased compared with healthy volunteers; in contrast, no miRNAs increased significantly (Sievers et al., 2012). Down-regulated miRNAs in B cells of MS patients include miR-25, miR-106b, miR-93, miR-19b, and miR-181a, which are essential for B cell development (Liang and Qin, 2009). Examination of T_reg_ cells in MS (De Santis et al., 2010) has identified differential expression of 23 miRNAs compared with healthy controls. Of particular interest, miR-106b and miR-25 were among the significantly decreased miRNAs, both of which modulate TGF-β signaling (Petrocca et al., 2008).

## miRNA DYSREGULATION IN CNS LESIONS

Approximately 60% of miRNAs identified to date are expressed in the CNS, many of which are highly enriched or specific to the CNS. Fine-tuning by miRNAs is key for proper gene expression during CNS development and normal function in adulthood, whereas abnormal miRNA expression is associated with multiple CNS disorders. Specifically, miRNA regulation is essential for neurogenesis, neurite outgrowth, oligodendrocyte differentiation, and myelin maintenance (Vo et al., 2005; Krichevsky et al., 2006; Shin et al., 2009; Dugas et al., 2010; Zhao et al., 2010). Disruption of miRNA function has been implicated in the pathogenesis of diseases such as amyotrophic lateral sclerosis, Alzheimer’s disease, and MS (Junker et al., 2009; Williams et al., 2009; Noorbakhsh et al., 2011).

To date, few studies have profiled miRNA expression in CNS tissue from MS patients, animal models, and inflammatory lesions of other autoimmune diseases. Analyses of CNS tissue that examined the profile of miRNA expression found in active lesions, inactive lesions, and normal appearing white matter (NAWM), together provide a signature of 50 miRNAs that are up-regulated and 30 miRNAs that are down-regulated in MS in comparison to healthy subjects (Junker et al., 2009; Noorbakhsh et al., 2011). Interestingly, there is little overlap between miRNAs that are dysregulated in different MS lesions and NAWM, suggesting that MS pathophysiology is heterogeneous at the level of miRNA control of gene expression.

Modified miRNA profiles detected in MS brain tissue may derive from alterations in neural cells or the immune cells that have infiltrated into the CNS parenchyma. Efforts have therefore been made to assign dysregulated miRNAs to specific cell types (**Figure 1**). Laser capture micro-dissection of active lesions has revealed that many up-regulated miRNAs are expressed by T cells, B cells, macrophages, and astrocytes (Junker et al., 2009). Comparison of the miRNA signatures of active lesions with healthy astrocytes, oligodendrocytes, and axons, reveals that the 10 most up-regulated miRNAs in active lesions are induced in astrocytes by IL-1β treatment (Lau et al., 2008; Junker et al., 2009; Letzen et al., 2010; Moser and Fritzler, 2010; Natera-Naranjo et al., 2010; Tarassishin et al., 2011). Among these highly expressed miRNAs, miR-155 is also dysregulated in NAWM and in the brains of EAE animals (Noorbakhsh et al., 2011; Lescher et al., 2012; Zhu et al., 2012). Interestingly, miR-155 expression is also increased in inflammatory lesions of patients and animal models of rheumatoid arthritis and lupus, suggesting that miR-155 dysregulation may have a broad role in promoting inflammation (Zhu et al., 2012).

Analysis of predicted targets of miR-155 combined with mRNA profiling of MS lesions has suggested several proteins that may be regulated by miR-155 in the CNS of MS patients. Among these candidates, CD47, ARK1C1, and ARK1C2 have been experimentally validated as miR-155 targets (Junker et al., 2009; Noorbakhsh et al., 2011). CD47 is a ubiquitously expressed protein that inhibits macrophage phagocytosis and functions as a “don’t eat me” message; miR-155-mediated down-regulation of CD47 is thought to release macrophages inhibition and thereby promote myelin breakdown (Junker et al., 2009). ARK1C1 and ARK1C2 are different isoforms of an enzyme encoding a 3-α-hydroxysteroid dehydrogenase activity that is essential to the synthesis of neurosteroids in the brain. Neurosteroid synthesis is impaired in MS, and the underlying mechanism is thought to at least partially depend on miR-155 and its negative regulation of ARK1C enzymes (Noorbakhsh et al., 2011). miR-155 down-regulates levels of astrocyte-derived cytokines produced *in vitro* in response to IL-1β/IFN-γ, suggesting a role for miR-155 promoting cytokine production in the brain. Interestingly, this effect is mediated by the down-regulation of SOCS1, a negative regulator of cytokine production that is directly targeted by miR-155 (Tarassishin et al., 2011).

miR-146a also exhibits elevated levels of expression in active MS lesions, EAE, and other autoimmune diseases (Junker et al., 2009; Lescher et al., 2012; Zhu et al., 2012). This NF-κβ-induced miRNA was initially shown to constitute an important negative feedback loop regulating the innate immune response in monocytes by targeting of IRAK1 (Taganov et al., 2006). However, more recent data have suggested that miR-146a-mediated down-regulation of IRAK1 is associated with the reinforcement of IRAK2-induced activation of NF-κβ and a sustained inflammatory response in human astroglial cells (Cui et al., 2010). Further support for a pro-inflammatory role of miR-146a in the brain is the evidence that miR-146a down-regulates the expression of complement factor H, a negative regulator of brain inflammation (Lukiw et al., 2008).

Among down-regulated miRNAs, miR-23b levels are decreased in MS inactive lesions, in the spinal cords of EAE mice and in inflammatory lesions associated with rheumatoid arthritis or systemic lupus erythematosus (Junker et al., 2009; Zhu et al., 2012). Findings derived from transgenic animal models indicate that miR-23b overexpression delays the onset of EAE and alleviates disease severity. Remarkably, miR-23b transgenic mice that have been irradiated and transferred with bone marrow of wild-type mice display the same phenotype, suggesting that during the normal course of EAE, miR-23b must be down-regulated specifically in brain-resident cells. Interestingly, IL-17, a pro-inflammatory cytokine, down-regulates miR-23b in astrocytes cultured *in vitro*. Target prediction and microarray analysis following miR-23b overexpression revealed a list of potential targets that includes many pro-inflammatory genes. Among these, TAB2, TAB3, and IKK-α were experimentally validated as miR-23b targets. Notably, these genes are up-regulated during EAE and mediate IL-17-, TNF-α-, and IL-1β-induced activation of NF-κβ. Together, these results provide evidence that miR-23b is a negative regulator of autoimmune inflammation that is down-regulated in MS lesions (Zhu et al., 2012).

miRNAs miR-214 and miR-23a are among the few miRNAs that are dysregulated in both active and inactive lesions (Junker et al., 2009). Additionally, miR-214 is up-regulated in lesions associated with other autoimmune diseases, whereas miR-23a is elevated in the spinal cord of mouse and non-human primate EAE (Lescher et al., 2012; Zhu et al., 2012). Elevation of miR-214 and miR-23a in MS lesions may reflect ongoing remyelination: miR-214 is up-regulated in oligodendrocytes during differentiation and miR-23a overexpression promotes oligodendrocyte differentiation by targeting and down-regulating lamin B (Lau et al., 2008; Lin and Fu, 2009).

In inactive MS lesions, miR-219 and miR-338-5p are the most down-regulated miRNAs (Junker et al., 2009). Expression of these miRNAs was detected in human oligodendrocytes isolated from the adult brain (de Faria et al., 2012). Importantly, regulation of the enzyme ELOVL7 by miR-219 is essential for myelin maintenance and axonal integrity in the adult mouse CNS (Shin et al., 2009). Lower levels of these miRNAs detected in inactive MS lesions may simply be due to the oligodendrocyte cell death that has occurred at these sites. In contrast, miR-219 and miR-338 are increased in NAWM of MS patients, along with other miRNAs that are normally up-regulated during oligodendrocyte differentiation. Moreover, five miRNAs that are normally down-regulated during oligodendrocyte differentiation are also down-regulated in the NAWM of individuals with MS (Lau et al., 2008; Letzen et al., 2010; Noorbakhsh et al., 2011). Such a profile, together with both miR-219 and miR-338-5p targeting inhibitors of oligodendrocyte differentiation such as Sox6, Hes5, and Zfp238 (Dugas et al., 2010; Zhao et al., 2010), suggest that oligodendrocyte precursors are differentiating in NAWM, presumably for the purpose of remyelination. In addition, miR-338 may have a role in MS pathology, as it is found in healthy axons and targets neurosteroid synthesis enzymes ARK1C1 and ARK1C2 (Natera-Naranjo et al., 2010; Noorbakhsh et al., 2011).

Comparison of miRNAs signatures reveals that 50% of the miRNAs that are up-regulated in MS in humans are also up-regulated during EAE. In addition, five of the six miRNAs most up-regulated during EAE are also up-regulated in active MS lesions (Junker et al., 2009; Noorbakhsh et al., 2011; Lescher et al., 2012; Zhu et al., 2012). In contrast, miR-326, the third most up-regulated miRNA in MS is only slightly increased during EAE, and miR-650, the top most up-regulated miRNA in MS is human-specific (Junker et al., 2009; Lescher et al., 2012). Even though this mechanism of up-regulation is not conserved, these miRNAs appear to be relevant to the inflammatory response. miR-650 is also up-regulated during rheumatoid arthritis and miR-326 directly targets CD47, and as such, may be involved in the inhibitory release of macrophages (Junker et al., 2009; Zhu et al., 2012).

In summary, miRNA dysregulation in MS lesions appears to be a significant contributor to the molecular mechanism that underlies inflammation in MS. This is best exemplified by the up-regulation of pro-inflammatory miRNAs such as miR-155 and the down-regulation of anti-inflammatory miR-23b; these changes occur specifically, but not exclusively, in astrocytes. It is also clear that alterations in the normal miRNA signature reflect both demyelination and CNS attempts to remyelinate degenerating axons. This is perhaps best illustrated by the up-regulation of miR-219-5p, miR-338, and miR-23a in the NAWM: miRNAs that promote oligodendrocyte differentiation. Although yet to be demonstrated, miRNA signatures characteristic of degenerating axons and activated microglia may also indicate miRNA dysregulation and may identify novel miRNAs that contribute functionally to MS.

## DISCUSSION AND PERSPECTIVES

Profiles of blood cells and CNS lesions of MS patients have revealed that miRNA expression is dysregulated in MS. Accumulating evidence indicate that such dysregulation is a relevant aspect of the disease pathology and that disruption of miRNA expression underlies biochemical alterations that occur in MS. Whether this abnormal miRNA expression contributes to the initiation of the disease has yet to be determined. It is possible that dysregulation of pro-inflammatory miRNAs such as miR-326 and miR-155 triggers early events that lead to inflammation. miRNAs miR-326 and miR-155 modulate T cell polarization and may contribute to CD4^+^T cell-mediated autoimmunity (Du et al., 2009; O’Connell et al., 2010). In addition, elevated levels of these miRNAs contribute to inflammation in the brain by releasing CNS-invading macrophages from inhibition (Junker et al., 2009). Further investigation is necessary to evaluate the role of these and other miRNAs in the initiation of MS.

The involvement of miRNAs in the pathology of MS suggests that modulators of miRNA expression or function may be used as disease-modifying therapeutics. In fact, anti-miR-326 and anti-miR-155 injections reduce the severity of EAE (Du et al., 2009; Murugaiyan et al., 2011) and EAE mice that are genetically modified to overexpress miR-23b also develop a milder form of disease (Zhu et al., 2012). A miRNA therapy would have to overcome potential side effects arising from the ability of miRNAs to regulate multiple targets in diverse cell types. Packaging into lipid vesicles containing appropriate receptors could serve to specify miRNA delivery to the appropriate cell type and reduce off-target effects. Interestingly, endogenous miRNAs are transported in the blood, inside plasma membrane-derived vesicles, and exogenous miRNAs can be successfully delivered using HDL particles purified from the blood (Valadi et al., 2007; Vickers et al., 2011).

Investigating the functional consequences of altered miRNA expression in CNS lesions and immune cells of MS patients poses a large challenge given the multiple mRNAs targeted by any one miRNA. Combinations of miRNA and mRNA/protein signatures may represent a possible approach to overcome this difficulty. Given the intimate association of axons, myelin, and astrocytes, in addition to the cross-talk between the adaptive and innate immune systems, it is critical to understand how manipulating specific miRNAs affects the biology of individual cell types, and also how altering miRNAs impacts the overall CNS and immunity. Further investigating the consequences of miRNA dysregulation will contribute to a better understanding of the pathophysiology of MS.

## Conflict of Interest Statement

Amit Bar-Or has acted as a consultant for Bayer, Bayhill Therapeutics, Berlex, Biogen-IDEC, BioMS, Diogenix, Eli-Lilly, Genentech, GSK, Guthy-Jackson/GGF, Merck-Serono, Novartis, Ono, Roche, Teva Neuroscience, and Wyeth. Jack P. Antel has acted as a consultant for TEVA, NOVARTIS, Serono, Biogen, Sanofi, Genzyme, and as part of the Burroughs Welcome Medical Scientist Program board.
