# Erratum: microRNA dysregulation in multiple sclerosis

**DOI:** 10.3389/fgene.2013.00090

**Published:** 2013-05-21

**Authors:** Craig S. Moore, Omar De Faria, Jack Antel, Amit Bar-Or, Timothy E. Kennedy, Ajit Dhaunchak

**Affiliations:** ^1^Department of Neurology and Neurosurgery, The Montreal Neurological Institute and Hospital, McGill University Health Centre, McGill UniversityMontreal, QC, Canada; ^2^Neuroimmunology Unit, Montreal Neurological Institute and Hospital, McGill University Health Centre, McGill UniversityMontreal, QC, Canada; ^3^Program in NeuroEngineering, McGill UniversityMontreal, QC, Canada

To the Editor,

In our review article entitled “MicroRNA dysregulation in multiple sclerosis” published online on January 22nd, 2013, we have made an error that we would like to correct.

We request to change the following sentences in the review article:

“Examination of Treg cells in MS (De Santis et al., 2010) has identified differential expression of 23 miRNAs compared with healthy controls. Of particular interest, miR-106b and miR-25 were among the significantly decreased miRNAs, both of which modulate TGF-β signaling (Petrocca et al., 2008).”

To

“Examination of Treg cells in MS (De Santis et al., 2010) has identified differential expression of 23 miRNAs compared with healthy controls. Of particular interest, miR-106b and miR-25 were among the significantly increased miRNAs, both of which modulate TGF-β signaling (Petrocca et al., 2008).”

We would also like to apply this change in a revised figure (Figure 1) that reflects the correct directionality of the altered miRNA expression in Treg cells in MS.

**Figure 1:**
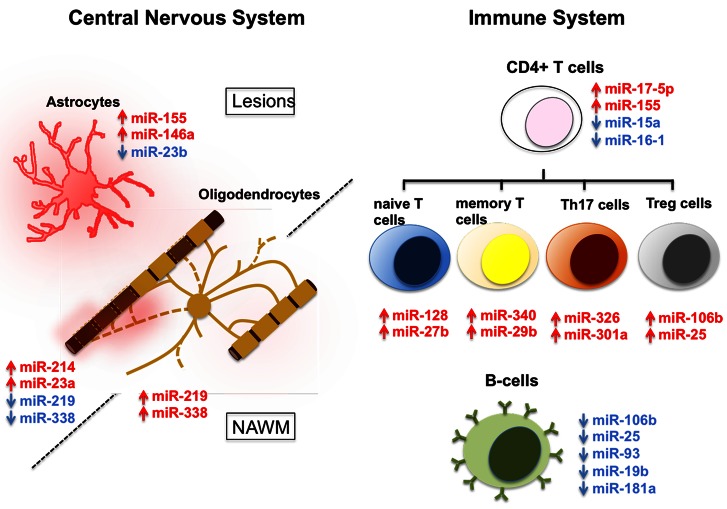
**MicroRNA (miRNA) dysregulation in CNS lesions and immune cells of MS patients.** miRNAs that are up- (red) and down-regulated (blue) in CNS lesions, NAWM, or immune system of MS patients have been assigned to specific cell types. Dysregulation is associated with astrocytes and oligodendrocytes in the CNS and naïve T cells, memory T cells, Th17 cells, Treg cells, and B cells in the immune system. Note that while in most cases miRNA dysregulation has been specifically detected in the mentioned cell types, miRNAs that are here assigned to oligodendrocytes, were done so exclusively on the basis of their relevance to normal oligodendrocyte biology.

We apologize to the readers of *Frontiers in Genetics* and to the authors of the De Santis et al., manuscript, for this error that was not corrected during the review process of our manuscript.
